# Factors released by low and high-LET irradiated fibroblasts modulate migration and invasiveness of pancreatic cancer cells

**DOI:** 10.3389/fonc.2022.1003494

**Published:** 2022-10-12

**Authors:** Alexandra Charalampopoulou, Amelia Barcellini, Mario Ciocca, Riccardo Di Liberto, Francesca Pasi, Marco Giuseppe Pullia, Ester Orlandi, Angelica Facoetti

**Affiliations:** ^1^ Research and Development Department, National Center for Oncological Hadrontherapy (CNAO), Pavia, Italy; ^2^ Clinical Department, National Center for Oncological Hadrontherapy (CNAO), Pavia, Italy; ^3^ Medical-Physics Department, National Center for Oncological Hadrontherapy (CNAO), Pavia, Italy; ^4^ Department of Medical Physics, IRCCS Policlinico San Matteo Foundation, Pavia, Italy; ^5^ Medical Oncology Unit, IRCCS Policlinico San Matteo Foundation, Pavia, Italy

**Keywords:** tumor microenvironment, pancreatic cancer, low-LET, high-LET, carbon ions, migration, invasion

## Abstract

**Introduction:**

Radiotherapy represents a major treatment option for patients with pancreatic cancer, however, its benefits remain limited also due to the ability of cancer cells to migrate to the surrounding tissues. Low-LET ionizing radiation is well known to promote tumor cell migration and invasion, nevertheless, little data provided by studies using high-LET radiation has led to ambiguous findings. What is hypothesized to be fundamental in the modulation of migration of tumor cells exposed to ionizing radiation is the influence of the microenvironment. Therefore, the properties of cells that populate the tumor stroma cannot be ignored when studying the influence of radiation on the migratory and invasive capacity of cancer cells. This is especially important in the case of pancreatic malignancies that are characterized by an abundance of stromal cells, including cancer-associated fibroblasts, which are known to orchestrate the cross-talk with tumor cells.

**Aim:**

The current study aims to investigate whether the presence of factors released by irradiated fibroblasts affects the migratory and invasive capacity of pancreatic cancer cells exposed to different doses of photons or C-ions.

**Materials and methods:**

AsPC-1 and AG01522 cells were irradiated with the same dose of photons or C-ions at room temperature. Through Boyden chamber assay, we tested whether factors secreted by irradiated fibroblasts may influence tumor cell migration, while the invasiveness of AsPC-1 cells was assessed using matrigel precoated inserts in which medium collected from non-irradiated (0 Gy), photon and C-ion irradiated fibroblasts, was added. Data were analyzed by Student t-test using GraphPad software. The mean ± s.d. was determined with a significance level of p<0.05.

**Results:**

In the presence of conditioned medium collected from 1 Gy and 2 Gy photon irradiated fibroblasts, the number of migrated tumor cells increased (P<0.0360, P<0.0001) but decreased at 4 Gy dose (P<0.002). There was a trend of reduction in migration (P<0.0460, P<0.038, P<0.0024, P<0.0002), as well as a decrease in invasiveness (P<0.0525, P<0.0035, P<0.0868, P<0.0310) after exposure to 0.5 Gy, 1 Gy, 2 Gy and 4 Gy of C-ions.

**Conclusions:**

The presence of irradiated fibroblasts affected the invasiveness capability of pancreatic cancer cells, probably by the reciprocal release of soluble factors whose production is differently modulated after high or low-LET radiation. Understanding the effects of irradiation on the metastatic potential of pancreatic cancer cells is of utmost importance for improving the outcome and tailoring the therapeutic approach. This challenging scenario requires a continuous and multidisciplinary approach that involves clinicians together with researcher experts in oncological and radiation treatment. In the last years, including preclinical experiences in a multidisciplinary approach has proved to be a winning strategy in clinical oncological research.

## Introduction

Pancreatic cancer is considered a “big killer” due to its local aggressive behavior and rapid metastasis. This kind of tumor is regarded as radioresistant histology due to the significant amount of hypoxic cells, and even though radiotherapy is advised in various clinical applications, the benefits are still unsatisfactory (1, 2). To overcome pancreatic cancer's intrinsic radioresistance, a dose-escalation radiation technique is required. In any case, radiation oncologists face a challenge in delivering a high dosage to the target because the pancreas is surrounded by healthy organs in motion and displays a high radiation sensitivity (i.e. duodenum, stomach, small bowel). In this context, particle therapy, particularly carbon ions (C-ions) radiation, appears to have promising benefits (3–5).. Contrary to expectation, radiotherapy has been proven to be able to act not only locally but can also provide a systemic effect.

Indeed, preclinical studies showed that ionizing radiation induces a high number of modifications, both in tumor cells and in surrounding stroma, fundamental in achieving the therapeutic goal and its well-known cytocidal effect (6, 7). In recent years, several experimental studies have demonstrated that ionizing radiation may promote migration and invasion of different tumor cells, highlighting the importance of interactions between tumor cells and their microenvironment (8). Regarding pancreatic tumors, it has been reported that, after photon irradiation, interactions between pancreatic cancer cells and surrounding fibroblasts play an important role in tumor progression and invasion (9, 10). C-ion irradiation in contrast to photons produces elevated ionization densities within individual tracks causing complex and unrepairable DNA damages which induce cell death. Different physical and radiobiological properties of C-ion irradiation have led researchers to hypothesize that hadrontherapy may have a different outcome in the radiation-induced mobility of cancer cells. Indeed, several authors reported that C-ions may produce a significant benefit by decreasing the migration and invasiveness of cancer cells *in vitro* and *in vivo*, although these results have been conflicting for certain cell lines (8, 11, 12). The present short report aims to determine whether irradiated fibroblasts’ released factors can influence the motility of AsPC-1 pancreatic cancer cells after exposure to different doses of photons or C-ions.

## Materials and methods

### Cell lines

Human pancreatic adenocarcinoma AsPC-1 cells were purchased from the Istituto Zooprofilattico Sperimentale della Lombardia e dell’Emilia Romagna (IZSLER, Italy) and cultured in RPMI 1640 medium containing glutamine and supplemented with 1% penicillin and streptomycin, 1% of sodium pyruvate, and 20% of fetal bovine serum (FBS). Low-passage human fibroblasts AG01522 were maintained in Minimum Essential Medium (MEM) alpha-modified supplemented with 2% L-glutamine, 1% penicillin and streptomycin, and 20% of fetal bovine serum (FBS). All cells were grown at 37°C in a humidified atmosphere containing 5% CO2 and split using 10% trypsin when reached confluency. For migration chamber assays 10% FBS medium was used in the upper inserts. All media, supplements, and chemicals were purchased from Sigma-Aldrich (St. Louis, MO, USA).

### Irradiations

AsPC-1 and AG01522 cells were irradiated with X-rays using a Siemens Primus linac at a dose rate of 2 Gy/min at the oncological radiotherapy department (San Matteo) in Pavia. Cells were grown in T25 flasks horizontally positioned above a 1.5 cm-thick layer of Plexiglas to ensure electronic equilibrium and irradiated with 0 Gy, 0.5 Gy, 1 Gy, 2 Gy and 4 Gy. C-ion radiations were performed with the clinical beams at National Center for Oncological Hadrontherapy (CNAO) in Pavia. A 6 cm Spread Out Bragg Peak (SOBP) in depth was generated (energy range: 246 -312 MeV/u) and the cells were positioned at the center of the SOBP (LET average of 75 KeV/µm). Fulfilled T12.5 flasks were vertically irradiated in a water phantom. All irradiations were performed at room temperature.

### Migration and invasion assays

Irradiated AsPC-1 cells were harvested from flasks and seeded with 10% FBS medium in duplets on inserts (VWR International, PA, USA). In the lower compartments (VWR International, PA, USA) 1.5 ml of 20% fresh complete medium was added. To evaluate the influence of factors released by irradiated AG01522 cells, 1.5 ml of 0.2 micron-filtered conditioned medium collected 1 hour after irradiation from irradiated cells was placed in the lower well as a chemo-attractant; all plates testing for migration assay incubated for 48 hours. Subsequently, cells on the upper surface of the inserts were removed by scrubbing with a cotton-tipped swab. Migrating cells in the lower surface of the membranes were washed twice with PBS, fixed with 70% cold ethanol for 3 minutes, washed and stained for 10 minutes with Gentian Violet solution. BioCoat Matrigel Invasion Chamber 8.0µm PET Membrane porous filters (Corning, MA, USA) were used as a barrier in Boyden chambers to test the invasive ability of irradiated AsPC-1 when conditioned medium from normal irradiated fibroblasts was placed in the lower well as an attractant. Duplicates were prepared for each examined condition and experiments were performed three times.

### Statistical analysis

Data were analyzed by Student t-test using GraphPad software. The mean ± s.d. was determined with a significance level of p<0.05.

## Results

### Migration assay

**Figure 1:**
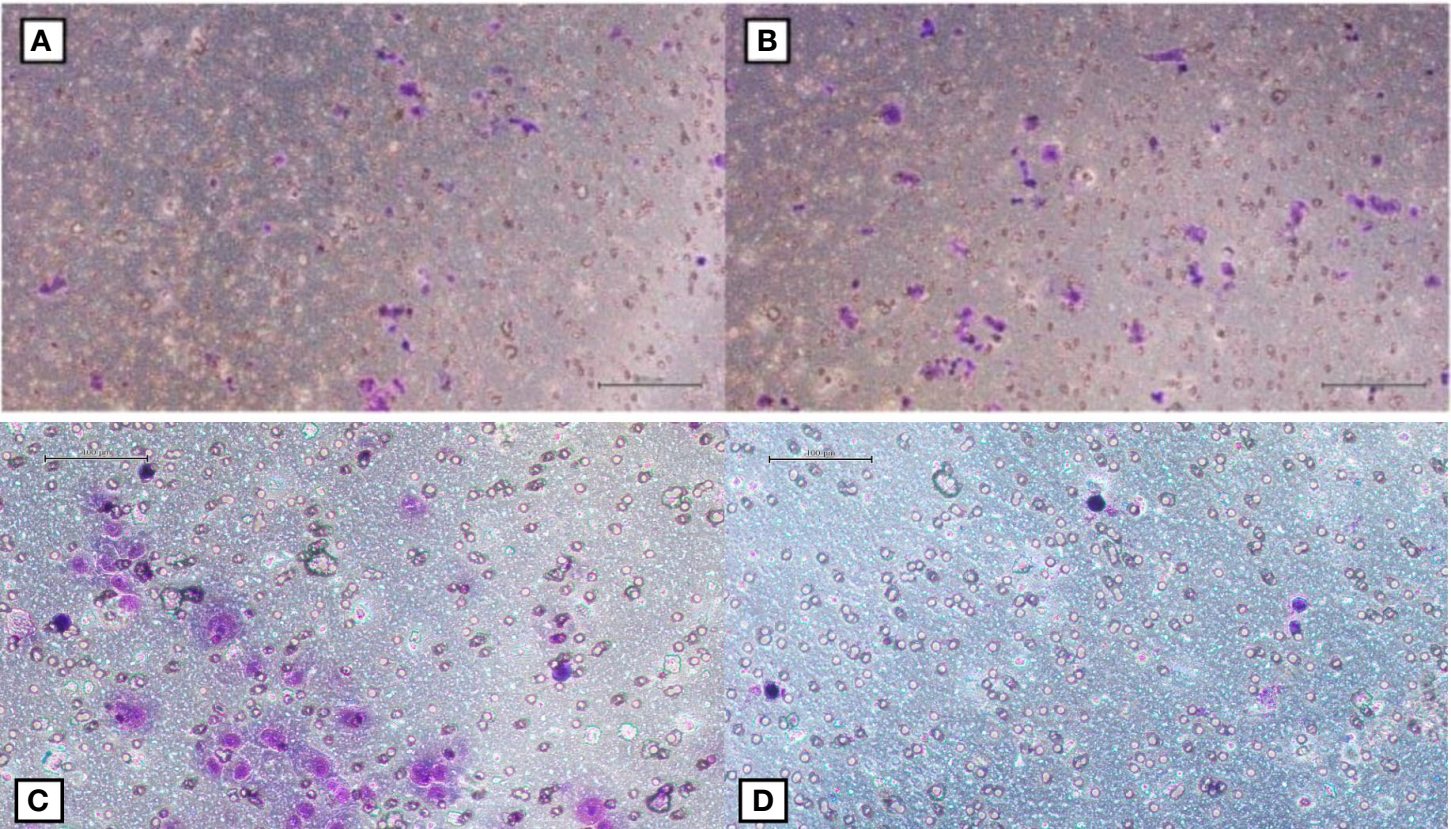
**(A, B)** Migrated AsPC-1 cells after the exposure of fibroblasts to 1 Gy and 2 Gy of photons respectively; **(C, D)** Migrated AsPC-1 cells after the exposure of fibroblasts to 1 Gy and 2 Gy of C-ions respectively.

**Figure 2:**
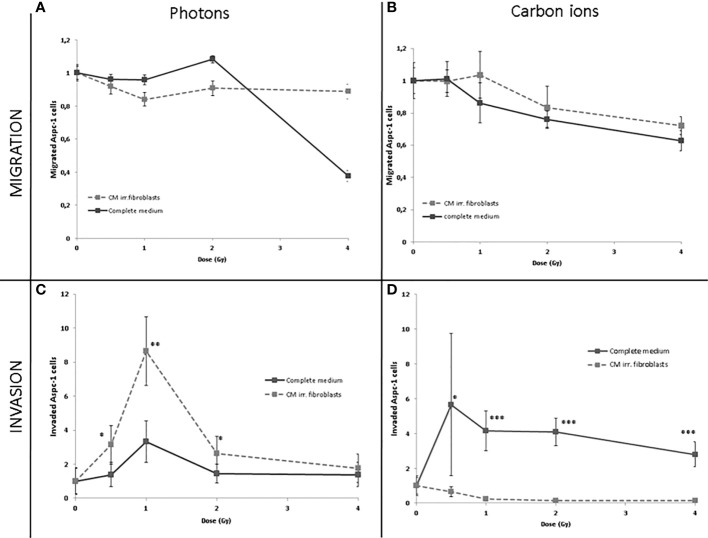
Migrated **(A, B)** or invaded **(C, D)** AsPC-1 cells photon **(A, C)** or C-ion **(B, D)** irradiated toward complete fresh medium collected from irradiated normal fibroblasts. Data are normalized to control condition and are presented as mean ± SDs of triplicate experiments. * statistically significant; ** very statistically significant; *** extremely statisically significant.

AsPC-1 cells were assayed for the effect of photon or C-ion irradiation on their migration ability. Through Boyden chamber assay, we tested whether factors secreted by irradiated normal fibroblasts may influence tumor cell migration (**Figures 1A–D**). In the presence of conditioned medium collected from 1 Gy and 2 Gy photon irradiated fibroblasts, the number of migrated tumor cells increased (P<0.0360 and P<0.0001, respectively) while it dramatically decreased when derived from a 4 Gy dose (P<0.002). In contrast, C-ion suppressed the migration in a statistically significant way compared to the control medium (P<0.0460, P<0.038, P<0.0024, P<0.0002) after exposure to 0.5 Gy, 1 Gy, 2 Gy and 4 Gy (**Figures 2A, B**).

### Invasion assay

Photon irradiation increased the ability of AsPC-1 cells to invade the matrigel coat. Analysis by radiation doses showed that among the tested doses, the highest increase was observed with medium corresponding to 1 Gy (P<0.0360). We also sought to test the effects of irradiation in medium from 0.5 Gy- and 2 Gy -irradiated fibroblasts and we found that it chemoattracted the tumor cells more efficiently (P<0.0494 and P<0.0466, respectively) than fresh complete medium (P<0.0559). Conversely, medium collected from C-ion-irradiated fibroblasts determined a strong decrease in invasiveness bringing the number of invading pancreatic cells to almost zero after exposure to 0.5 Gy, 1 Gy, 2 Gy and 4 Gy(P<0.0525, P<0.0035, P<0.0868, P<0.0310) (**Figures 2C, D**).

## Discussion

In the present *in vitro* study, we investigated whether the presence of factors released into the medium by irradiated fibroblasts can modulate the ability of tumor cells to migrate and invade. We also evaluated whether the type of radiation influenced this response since the role of normal surrounding cells like fibroblasts has yet to be investigated in the context of high-LET radiation-induced mobility of cancer cells. This issue is of utmost importance considering that understanding the effects of irradiation on the metastatic potential of cancer cells might help clinicians in improving the clinical outcome of cancer radiotherapy. Indeed, in recent years radiotherapy has been proven to be able to act not only locally but also to give a systemic effect. Indeed, preclinical studies showed that ionizing radiation not only interferes with the target of treatment but can revert immuno-suppressive factors within the tumor microenvironment, which (7) fundamental in achieving oncological control. Recent research has demonstrated that radiotherapy can alter the malignant behavior of surviving tumor cells by reducing or promoting their invasiveness or migration, depending on the form of radiation (low or high LET) and the investigated cell line (11). So far, C-ions were found to be effective in suppressing the invasive capability of AsPC-1 pancreatic cancer cells while increasing it in PANC-1 cells (11). Increased invasiveness of pancreatic cancer cells, when cocultured with fibroblasts, has already been reported (10). We previously stated that factors released by normal fibroblasts influence Aspc-1 cell migration and that the amoeboid-mesenchymal transformation of pancreatic cancer cells is accelerated in the presence of low or high LET irradiated fibroblasts (13)

In the current *in vitro* study, we found that photons failed to reduce the invasiveness of AsPC-1 and conversely C-ion irradiation suppressed the invasive potential of the cells. We observed that the increase in invasiveness following photons radiation was more evident with 1Gy dose, whereas high-LET radiation decreased the number of invading cells already irradiated with 1Gy dose, remaining steady after 2Gy and 4Gy. This finding suggests that on one hand the presence of irradiated fibroblasts affects the invasiveness capability of irradiated pancreatic cancer cells and, on the other, that the release of soluble factors is differently modulated after high or low-LET radiation. Indeed, while photons seemed to slightly increase the invasiveness of pancreatic cancer cells when co-cultured with normal fibroblasts, factors released from pancreatic cancer cells following C-ion radiation appeared to induce a stronger response in fibroblasts promoting inhibition of cancer cell invasion. This is not surprising given that high-LET radiations such as C-ions interact with cells and molecules differently than photons, activating different cellular pathways that lead to different, even opposing cellular responses.

Thus, a deeper investigation of the invasive capacity of different pancreatic cancer cells in response to co-culture with fibroblasts irradiated with sublethal doses of proton or C-ions would be important.

We have not yet examined whether normal fibroblasts’ released factors influence the motility of other pancreatic cell lines after exposure to different doses of C-ion radiation and further *in vitro* studies are required to confirm these findings. However, despite this limitation, this preliminary report is the first study showing that the presence of non-irradiated fibroblasts affected the invasiveness capability of irradiated pancreatic cancer cells differently according to the LET of radiation used.

From an oncological point of view, understanding these underlying mechanisms is an inestimable chance to clarify the unexpected effects of radiotherapy in order to provoke them leading to a new therapeutic era. Indeed, understanding the cross-talk between tumor and micro-environment after hadrontherapy might open a new area of research that focuses on the effect of particle therapy on systemic disease in addition to the immune system (14). The data generated in this study might serve as radiobiological basis for further preclinical and clinical studies leading to therapeutic and multidisciplinary strategies (15, 16) that counter highly invasive cell lines *via* specific inhibitors or C-ion therapy.

## Data availability statement

The raw data supporting the conclusions of this article will be made available by the authors, without undue reservation.

## Author contributions

AF and AB carried out cell migration and invasion assays, AF and AC performed data analysis. MC supervised the dosimetry and the carbon ion irradiations, RDL and FP assisted with irradiation and access to San Matteo Hospital. AF, AB, and AC conceptualized the study and wrote the first draft of the manuscript. MGP coordinated all the work. EO Critically revised manuscript. All authors contributed to the article and approved the submitted version.

## Funding

This work was financially supported by INFN grant ETHICS (“pre-clinical Experimental and THeoretical studies to Improve treatment and protection by Charged particleS”).

## Conflict of interest

The authors declare that the research was conducted in the absence of any commercial or financial relationships that could be construed as a potential conflict of interest.

## Publisher’s note

All claims expressed in this article are solely those of the authors and do not necessarily represent those of their affiliated organizations, or those of the publisher, the editors and the reviewers. Any product that may be evaluated in this article, or claim that may be made by its manufacturer, is not guaranteed or endorsed by the publisher.
